# Surrogate explanations for role discovery on graphs

**DOI:** 10.1007/s41109-023-00551-w

**Published:** 2023-05-26

**Authors:** Eoghan Cunningham, Derek Greene

**Affiliations:** 1 School of Computer Science, University College Dublin, Dublin, Ireland; 2 Insight Centre for Data Analytics, University College Dublin, Dublin, Ireland

**Keywords:** Role discovery, Node embedding, Explainable artificial intelligence

## Abstract

Role discovery is the task of dividing the set of nodes on a graph into classes of structurally similar roles. Modern strategies for role discovery typically rely on graph embedding techniques, which are capable of recognising complex graph structures when reducing nodes to dense vector representations. However, when working with large, real-world networks, it is difficult to interpret or validate a set of roles identified according to these methods. In this work, motivated by advancements in the field of explainable artificial intelligence, we propose surrogate explanation for role discovery, a new framework for interpreting role assignments on large graphs using small subgraph structures known as graphlets. We demonstrate our framework on a small synthetic graph with prescribed structure, before applying them to a larger real-world network. In the second case, a large, multidisciplinary citation network, we successfully identify a number of important citation patterns or structures which reflect interdisciplinary research.

## Introduction

Numerous approaches have been proposed in the literature for the task of structural role discovery on graphs, where nodes on a graph are divided into classes of *structurally similar* nodes called ‘roles’ (Rossi and Ahmed 2014). Early approaches in this area relied on graph sub-structures known as *graphlets* or *motifs* (Milo et al. 2002). In large graphs, where we wish to identify higher-order structural features, counting large graphlets is computationally expensive. Thus the majority of recent approaches to role discovery employ a representation learning technique known as node embedding. Node embedding refers to the task of mapping the nodes on a graph to some low-dimensional, dense vector space, which preserves important similarities between the nodes. In the context of role discovery, the embedding step is often called ‘role embedding’, and the similarities that are preserved between nodes should encode their structural features. While graphlet counts can be used as structural features in role embedding algorithms, more recent approaches prefer alternative features, like degree distributions (Ribeiro et al. 2017) and diffusion wavelet patterns (Donnat et al. 2018). These methods are designed to learn higher-order structural relationships than those that can be discovered by small graphlets. However, in many cases, these alternative approaches come at the cost of interpretability. When applied to graphs that are too large to be visualised reasonably, it is often difficult to understand the substantive meaning of a given set of structural roles.

Embedding methods for role analysis have previously been shown to be capable of grouping nodes into known roles or structures, such as those prescribed in synthetic graphs or those understood to exist in transport networks (Ribeiro et al. 2017; Donnat et al. 2018). However, it remains unclear as to how a set of discovered roles should be interpreted or validated when applied to real-world graphs with unknown structure. Moreover, different role discovery methods learn different sets of structural roles and, depending on the application, many or none of these clusterings may be valid. As such, it is critical that we can provide explanations for role discovery tasks, allowing us to validate discovered roles and compare alternative groupings generated by different methods.

The key contribution of our work is a new framework, surrogate explanation for role discovery (SERD), for explaining a set of discovered roles using *graphlet orbits* (Yaveroğlu et al. 2014), described in detail in Sect. “Methods”. For the purposes of motivation and validation, we demonstrate our proposed method on a small synthetic graph structure in Sect. “Demonstration/validation”. Later in Sect.  “Application” we apply the framework to a large, multidisciplinary real-world citation network to extract and interpret sets of structural roles. In this case study (initially presented in Cunningham and Greene (2023)), we demonstrate that our approach allows us to identify meaningful citation structures and patterns that are specific to interdisciplinary research.

## Related work

### Local graph structure and role embeddings

**Fig. 1:**
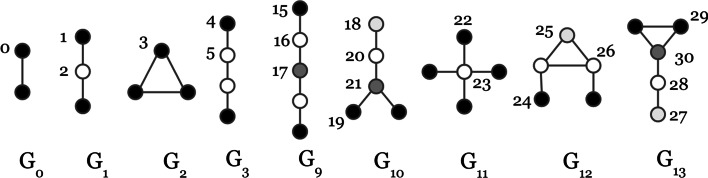
Examples of graphlets with 2, 3, 4, and 5 nodes. Nodes are shown in the same colour if they have equivalent position in the graphlet. Each of these postions is called an ‘orbit’. Graphlets and orbits are enumerated according to Pržulj (2007)

Research in the social sciences studied local graph structures using small graph patterns such as triads, cycles and stars (Moreno 1934). More recent computational research has elaborated upon these methods and proposed the term *network motif*—a subgraph pattern (or *graphlet*) which is significantly over-represented in a graph (Milo et al. 2002). Motif and graphlet counts represent powerful features for expressing graph structure, and have been employed in graph learning tasks such as node classification and anomaly detection (Cunningham et al. 2013). When using graphlet counts to describe nodes, it is more informative to consider the position in the graphlet at which the node occurs. Equivalent positions within graphlets are called *automorphism orbits* or simply ‘orbits’. Figure 1 illustrates a subset of graphlets with 2, 3, 4 and 5 nodes, and includes each of the distinct orbits on these graphlets, as they were enumerated by Pržulj (2007). A compelling example of the use of graphlet orbit counts in the analysis of complex networks is provided in Yaveroğlu et al. (2014).

*Role discovery* is the task of grouping nodes which share similar structural patterns in a graph into distinct classes (Rossi and Ahmed 2014). Many modern approaches to role discovery rely on graph embedding, where nodes are transformed into low-dimensional vector representations (Ahmed et al. 2019; Henderson et al. 2012; Ribeiro et al. 2017; Donnat et al. 2018). Specifically, graph embedding methods for the purpose of role discovery (often called “role embedding”) learn dense vector representations for nodes such that nodes that are structurally similar will be embedded nearby in the embedding space (i.e., will have similar representations). A clustering of the role embedding space thus represents a set of discovered roles, where each cluster should encode a distinct role. However, if this network cannot be visualised, it is difficult to interpret the resulting roles and to derive their meaning in a real-world context. Moreover, with numerous approaches to role embedding (e.g., Ribeiro et al. (2017); Ahmed et al. (2019); Henderson et al. (2012); Donnat et al. (2018)), and many possible clusterings of each embedding space, we require some approach to *explain* a set of discovered roles so that they can be compared and validated. In this work, we employ methods from the field of explainable artificial intelligence to provide downstream explanations of role discovery tasks using graph-orbit structures.

### Explanation via surrogate models

In recent years, much research has been conducted in the field of *explainable artificial intelligence* (XAI) towards the goal of understanding and explaining so-called ‘*black box*’ models (Adadi and Berrada 2018). One popular approach is to use a global surrogate model: “an interpretable model that is trained to approximate the predictions of a black box model” (Molnar 2020). As such, the decisions made by a previously uninterpretable system can be explained through interpretations coming from the surrogate model.

Some classification models (such a logistic regression models or decision tree-based classifiers) are interpretable by definition, as any input feature’s effect on the models classification can be measured (for example, using regression coefficients). However, many model-agnostic methods of interpretation have also been developed. Generally, these methods propose different means of perturbing the input data and exploring its effect on the models output. For example, *Partial Dependence Plots* (PDPs) (Friedman 2001) offer a graphical representation of a models prediction as a function of its arguments or inputs. Broadly speaking, a PDP shows how a given feature influences the prediction of a model, on average, for every value that it can take. The *Accumulated Local Effects* plot (ALE) (Apley and Zhu 2020) provides a similar illustration of a feature’s effect, with the key difference that it accounts for possible correlations between features, and as a result is more robust than the PDP in some applications.

In the context of network analysis, global surrogate models have been employed to provide feature-based explanations for communities detected on graphs (Sadler et al. 2021). Specifically, this work assessed the importance of different graph features using *permutation importance* (Breiman 2001), where the values for a feature are permuted throughout the dataset and the effect on model performance is measured to indicate its importance. While ample work exists toward interpreting and validating proximity-preserving graph embedding and community detection, similar research in validating and explaining role discovery is young. The few methods that have been proposed to date evaluate role embedding approaches according to their ability to group nodes with similar network properties such as PageRank centrality or clustering coefficient (Dehghan et al. 2023; Jin et al. 2021). In this work, we leverage advancements in the field of XAI to propose a method of explanation that highlights important sub-graph structures specific to the roles discovered on graph, and offer visual explanations for role discovery that are intended to augment existing methods of evaluation. We demonstrate this method first in Sect. “Demonstration/validation” on a small synthetic graph. Then, in Sect.  “Application”, we apply our methods to roles discovered on a large multidisciplinary citation network, in order to highlight some of the citation patterns that are emblematic of interdisciplinary research.

### Measuring interdisciplinarity

In light of the perceived importance of interdisciplinary research (IDR), many studies have been conducted that quantify article interdisciplinarity in an effort to identify relevant research trends and to explore their impact. The most widely-accepted methods for measuring interdisciplinarity assess *knowledge integration* or *knowledge diffusion* using citation information, thus measuring interdisciplinarity as some function of the balance, diversity, and dissimilarity of the disciplines identified in an article’s referenced papers (Porter and Rafols 2009; Rafols and Meyer 2010). Alternatively, some studies compute a similar score on the disciplines identified in an article’s *citing* literature, instead measuring IDR according to an articles impact/influence across multiple disciplines (Porter and Chubin 1985; Van Noorden 2015). A popular function for measuring IDR is the Rao-Stirling Diversity index (Stirling 2007)

1 $$\begin{aligned} D = \sum _{i,j (i\ne j)}p_ip_jd_{ij} \end{aligned}$$

where IDR is measured as the sum over all pairs of disciplines identified in a set of articles cited by (or citing) some focal paper. Here $p_i$ and $p_j$ denote the proportion of references to disciplines *i* and *j* respectively, while $d_{ij}$ refers to some precomputed distance between the disciplines.

To implement the metric in Eq. 1, or any of its many variants, each paper in a research corpus must be assigned to an explicit research topic or subject category, for which sources are numerous, inconsistent, and sometimes unavailable. Subject categories are most commonly assigned to papers according to the journals in which they are published. However, explicit categorisations for research papers, especially those assigned at a journal level, are problematic (Abramo et al. 2018; Milojević 2020). Such assignments rarely agree with underlying citation community structure (Porter and Rafols 2009). Moreover, scientific communities have been shown to be evolving rapidly (Rosvall and Bergstrom 2008) and the inconsistencies evident across many of these subject taxonomies (Shen et al. 2019) may confirm that no singular, correct categorisation exists. The limitations of prescribed, static, subject classifications necessitate a modern approach that identifies research disciplines—and indeed interdisciplinary research—according to research network structure. Such an approach may offer a dynamic view of interdisciplinarity, and may help to map emerging developments that do not fit any existing schema (Wagner et al. 2011).

There is evidence that interdisciplinary research can be identified in a scientific corpus according solely to the citation structure. Specifically, it has been shown that frameworks that encode the *structural role* of articles in a citation graph can predict interdisciplinary interactions more accurately than those that encode only the proximity between papers (Cunningham and Greene 2022). In light of this, later in Sect.  “Application” we explore the potential for modern graph learning methods to identify the citation structures associated with interdisciplinary research.

## Methods

**Fig. 2:**
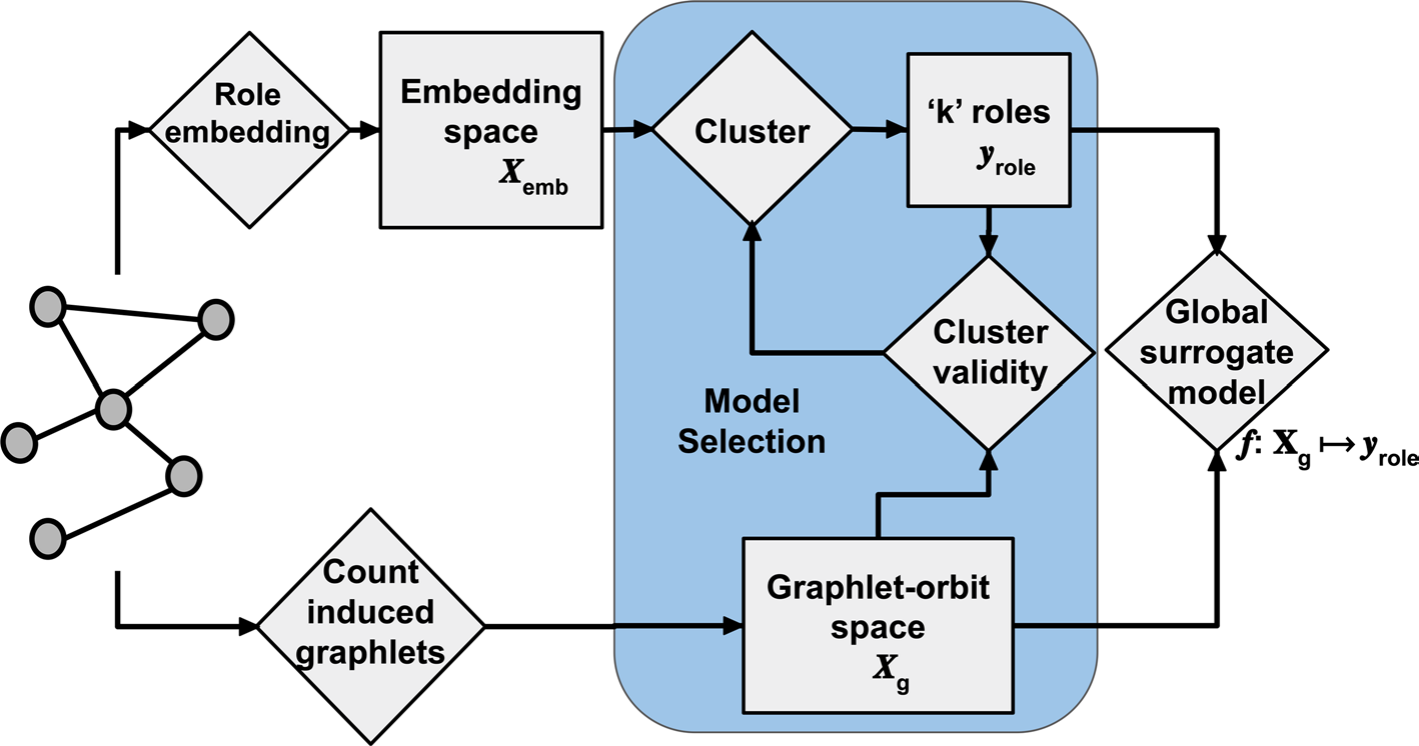
Overview of the complete workflow for the SERD framework to discover and evaluate roles

We now outline SERD, a general framework for uncovering and evaluating structural roles on a graph. An overview of the full process is illustrated in Fig. 2. We begin with a graph $G = (V,E)$, from which we wish to identify a set of discrete structural roles. We employ a role embedding algorithm to map each node $v \in V$ to an embedding space $X_{emb} = {\mathbb {R}}^{128}$, thus a clustering of $X_{emb}$ is considered a set of discovered roles $y_{role}$. Additionally, we represent the same nodes in the graphlet-orbit space $X_g = {\mathbb {Z}}^{73}$ derived from *G*, where each node *v* is represented by a *bag-of-orbits* vector $x^v = \{x_0^v, x_1^v,\ldots , x_{72}^v\}$, with $x_i^v$ denoting the number of times node *v* is involved in induced graphlet orbit *i*. We use a vocabulary of 73 orbits, i.e. we count all orbits on all graphlets of size 2 to 5 using the ORCA method (Hočevar and Demšar 2017). We refer to graphlets and orbits according to the enumeration provided in Pržulj (2007). The graphlet-orbit space is first used to validate a given set of roles that we identify in the graph. By clustering the embedding space, we group nodes into *k* discrete roles, which we can evaluate using cluster validity metrics calculated on the graphlet-orbit space (e.g., using the popular Silhouette index (Rousseeuw 1987)). Employing various role embedding algorithms and clustering methods, we identify a set of candidate clusterings (or roles) according to the separation they achieve in the graphlet-orbit space.

**Fig. 3:**
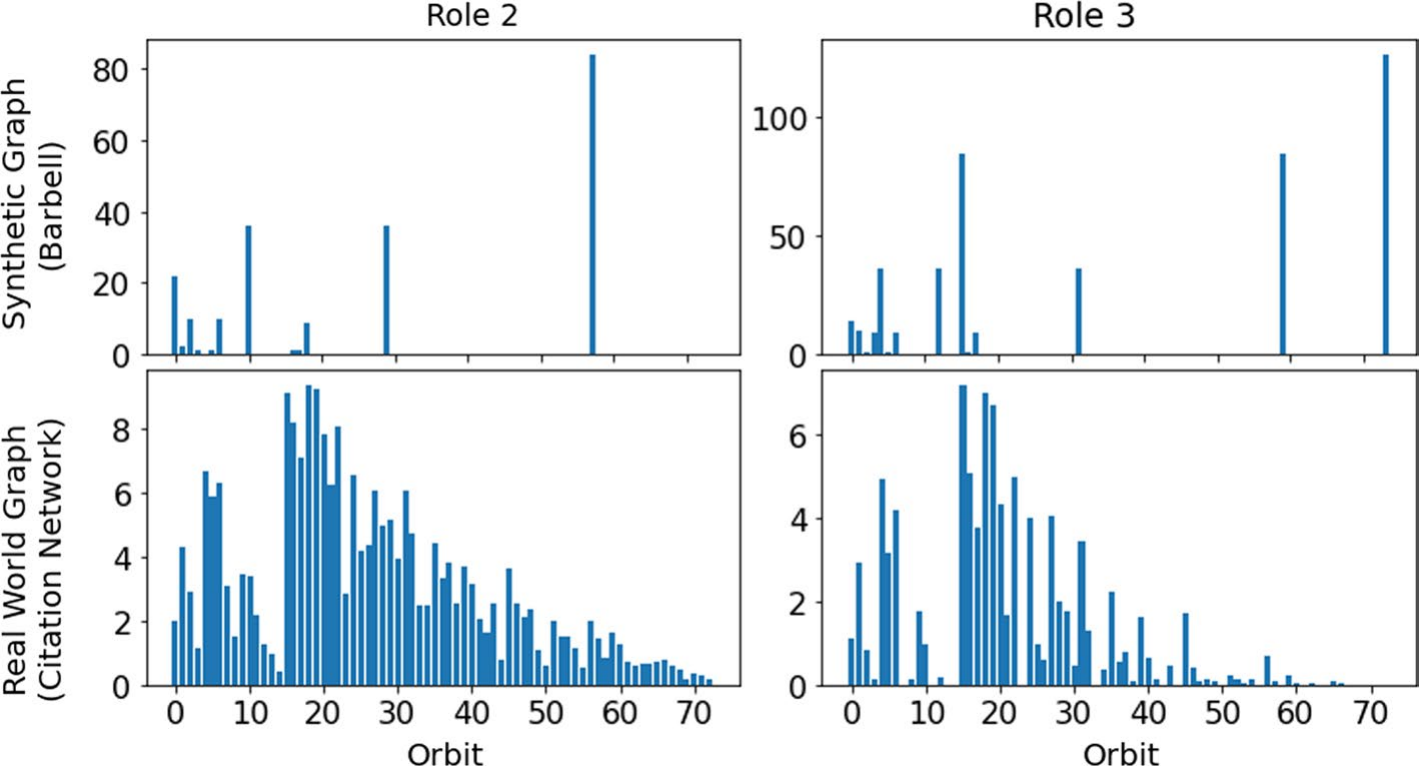
A visualisation of cluster centroids. Each bar plot shows the mean values of the graphlet-orbit counts for a particular cluster/role. The centroids in the top row are taken from a small synthethic barbell graph (described in Sect. “Demonstration/validation”), while the centroids in the bottom row are taken from a much larger graph, (the citation network described in the Sect. “Application”)

Graphlet-orbit counts are a powerful language for describing the structural features of nodes on a graph (Cunningham et al. 2013; Pržulj 2007). As such, we suggest that a clustering (according to node embeddings) which is valid in the graphlet-orbit space should offer a reasonable set of structural roles. Additionally, we employ graphlet orbits to interpret a set of discovered roles. For example, Fig. 3 illustrates the mean bag-of-orbits vector (or *cluster centroid*) for two pairs of roles, as discovered in a small synthetic graph and a large real world graph, (complete descriptions of these graphs are provided in Sects.  “Demonstration/validation” and “Application” respectively). Centroid analysis is commonly applied when evaluating and interpreting clusterings of feature-based data (Jain and Dubes 1988). For the context of clustering nodes based on graphlet orbit counts, centroids could be said to represent ‘*prototypes*’ of the respective roles, and can be evaluated and compared in an effort to understand the structures specific to those roles. In the case of the pair of roles discovered in the small synthetic graph (presented in the top row of Fig. 3), the centroids are sparse and the differences between the roles can be readily understood. However, in the case of a larger, real-world graph (e.g., the centroids presented in the bottom row of Fig. 3), the roles are more complicated and nuanced. As a result, the prototypes are far more difficult to parse and contrast. Further, depending on the shape of a cluster, its centroid may not provide a prototype that is close to a real case present in the data. In the case of these complex structures—and to a lesser extent in the case of the simple synthetic graphs—we require a method of interpreting role discovery that provides explanations that are ‘simple’ and ‘contrastive’ (Lombrozo 2007; Miller 2018). Due to these limitations of a centroid-based approach, we propose to explain role clusterings using surrogate models.

For a candidate clustering $y_{role}$, we fit a surrogate model $f:X_g\mapsto y_{role}$. By modelling the role assignment in the graphlet-orbit space, we can explore the feature importance and effect of the different graphlet orbits in role assignments, according to many *model-agnostic* explanation techniques from the field of XAI (Molnar 2020). Due to nature of graphlet structures, it is likely that correlations will exist between the features in $X_g$. Accordingly, we emphasise those methods of surrogate-based explanation that can account for such correlations. In Sect. “Demonstration/validation”, we demonstrate graphlet orbit-based explanation on a small synthetic barbell graph, using a simple logistic regression surrogate model. Following this, in Sect. “Application”, we apply graphlet orbit-based explanation using permutation importance (Breiman 2001) and accumulated local effects plots (Apley and Zhu 2020) to a large, real-world, citation network. As we will show, highlighting important or discriminatory orbits can offer a visual means of understanding the nature of a role in the graph.

## Demonstration/validation

**Fig. 4:**
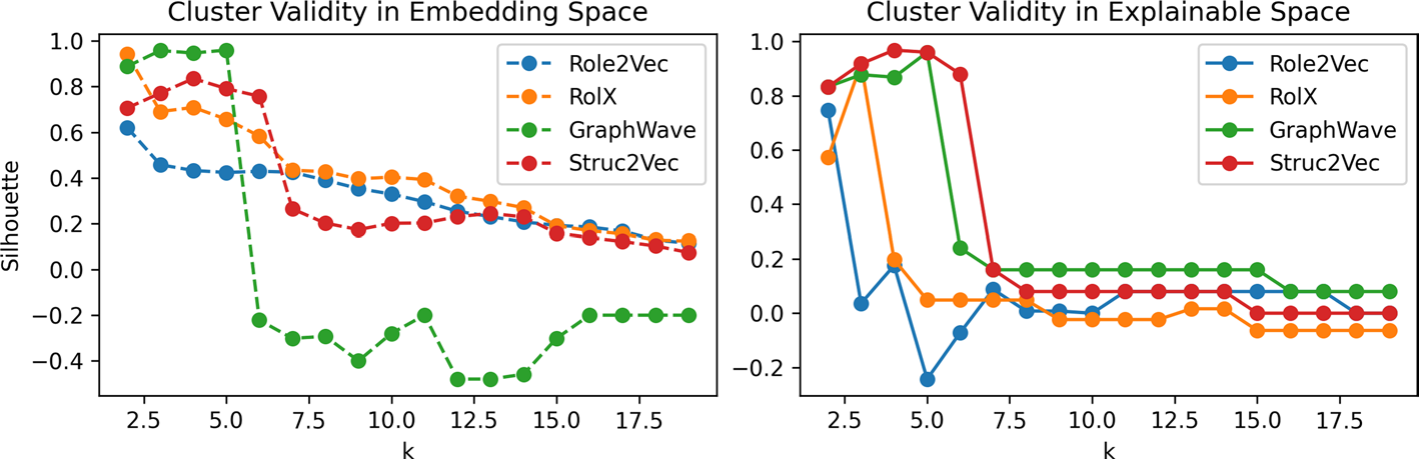
Role validity as calculated using the Silhouette measure for clusterings $k \in [2,19]$ generated using 4 role discovery methods. Validation scores were computed on the embedding spaces (left) and the explainable graphlet-orbit space (right)

Our proposed method is intended to be used to explain a set of discovered roles in the case where it is not feasible to visualise the graph in its entirety. In this section, for the purposes of demonstrating and validating our approach, we first present an application involving a small synthetic graph with pre-specified structure: the Barbell Graph. Barbell graphs are commonly used in the evaluation of Role Discovery methods (Ribeiro et al. 2017; Donnat et al. 2018; Ahmed et al. 2019), as they contain a prescribed number of structurally equivalent nodes (i.e., *roles*) that can be easily validated visually. Here, we consider a graph with two cliques of 10 nodes each, with a chain of 5 nodes between them. This structure contains one set of 5 *structurally equivalent* roles, but many smaller sets of *structurally similar* roles may also be considered valid.

### Role discovery

For each node in the barbell graph, we generate role embeddings according to 4 methods: (i) *Role2Vec* (Ahmed et al. 2019), (ii) *RolX* (Henderson et al. 2012), (iii) *GraphWave* (Donnat et al. 2018), and (iv) *Struc2Vec* (Ribeiro et al. 2017). We cluster these four embedding spaces using *k*-means clustering for each value of *k* in the range [2, 19]. The resulting clusterings represent different sets of discovered roles which we can interpret and compare using our method.

### Role interpretation

Figure 4 shows two plots of cluster validity for the roles discovered according to each of the 4 role discovery methods. Each set of roles represents the output of *k*-means clustering on one of the *embedding* spaces $X_{emb}$, where we assess the validity of the clustering using the Silhouette measure (Rousseeuw 1987) (presented on the left). Each clustering (or set of roles) is then transformed to the graphlet-orbit space $X_g$, where again, we measure the validity of the clustering using Silhouette score (presented on the right). Silhouette scores can take a value in the range [−1,1], where a high score represents dense, separate clusters, a score of 0 indicates an overlapping clustering, while a negative score indicates an incorrect clustering. The cluster validity (as measured in the embedding space $X_{emb}$), reports the extent to which nodes which have similar embeddings have been grouped together. However, in the context of a role discovery task, it is difficult to determine how well the role embedding step has preserved any structural similarity between the nodes. As such, we also measure the quality of all candidate sets of roles as clusterings in the graphlet-orbit space $X_g$. That is, we calculate Silhouette scores according to similarities and distances between nodes, when we describe them using their bag-of-orbits vectors. These scores are presented in Fig. 4 on the right-hand side. Comparing the two plots in Fig. 4, we can see cases where sets of roles which are equivalent (or at least equally valid in the graph-orbit space), have different silhouette scores in their respective embedding spaces, and many cases where roles which appear valid in the embedding space, give very poor separation in the graphlet-orbit space. In the case of this barbell graph, the *GraphWave* and *Struc2Vec* methods achieve roles that are the most valid in the graphlet-orbit space.

**Fig. 5:**
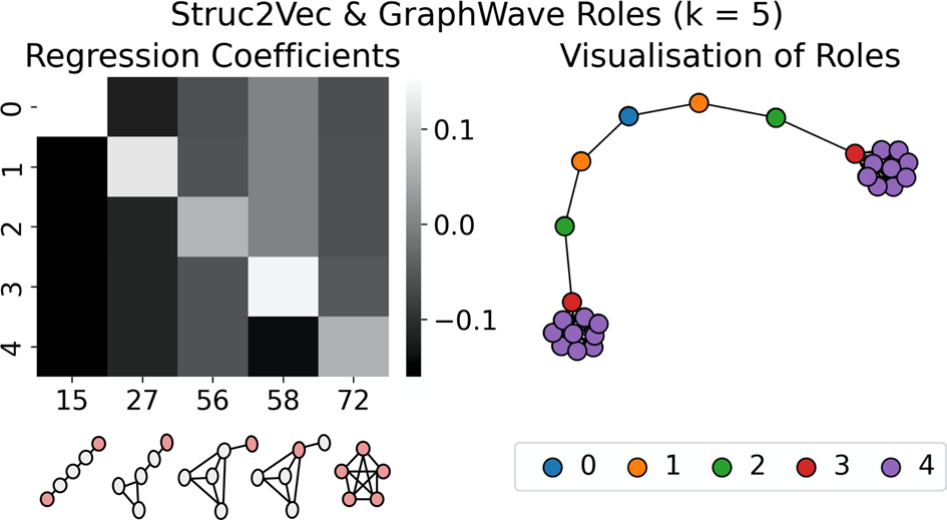
Structural explanation for 5 structurally-equivalent roles in the barbell graph. These roles were correctly identified by both *GraphWave* and *Struc2Vec* via *k*-means clustering of embeddings $(k = 5)$

We propose to interpret the structures specific to these roles (i.e., the different clusterings $y_{role}$) through *explanation* via a surrogate model $f: X_g \mapsto y_{role}$. That is, *f* is some function to classify nodes to their *GraphWave/Struc2Vec*-assigned roles according to their bag-of-orbits vectors, which record how often a node is involved in each position of each subgraph structure. For the purpose of this demonstration, we choose to model the role assignments in the graphlet-orbit space using a simple logistic regression with a LASSO. The *L*1 penalty in the LASSO regression serves to induce sparsity and ‘simplicity’ in the representation as it helps to identify those features that are most important to classification, and thus, the structures that best discriminate between the roles. Further, the LASSO regression acts as a method of feature selection and can account for any correlations that may exist between the orbit counts. At $k = 5$, *GraphWave* and *Struc2Vec* correctly identify the 5 sets of structurally equivalent roles in the barbell graph. Figure 5 shows the barbell graph on the right, with nodes coloured according to these role assignments, and the non-zero coefficients of *f* on the left in a heatmap. Thus, the important features are shown as the columns of the heatmap. Each column is labelled with an illustration of its associated graphlet-orbit.

**Fig. 6:**
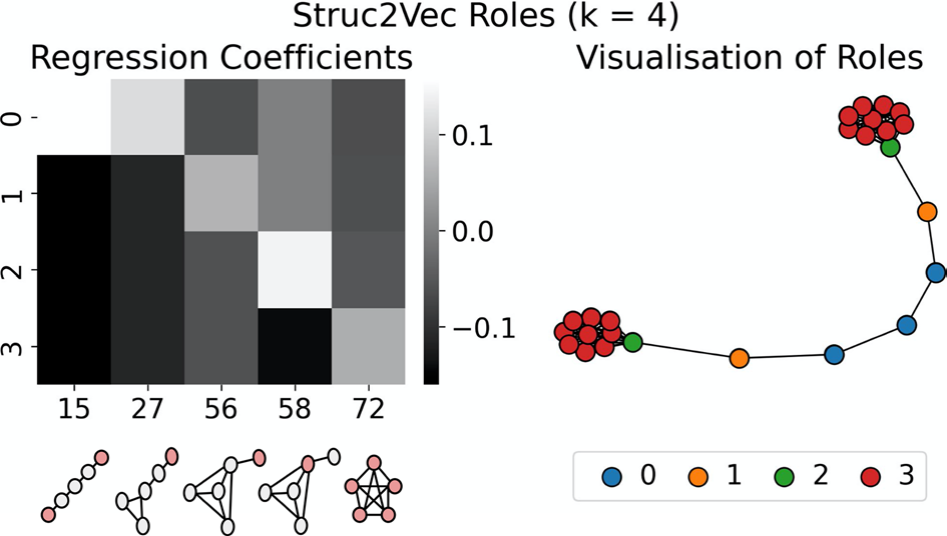
Structural explanation for role discovery via *k*-means clustering of *Struc2Vec* embeddings $(k = 4)$

**Fig. 7:**
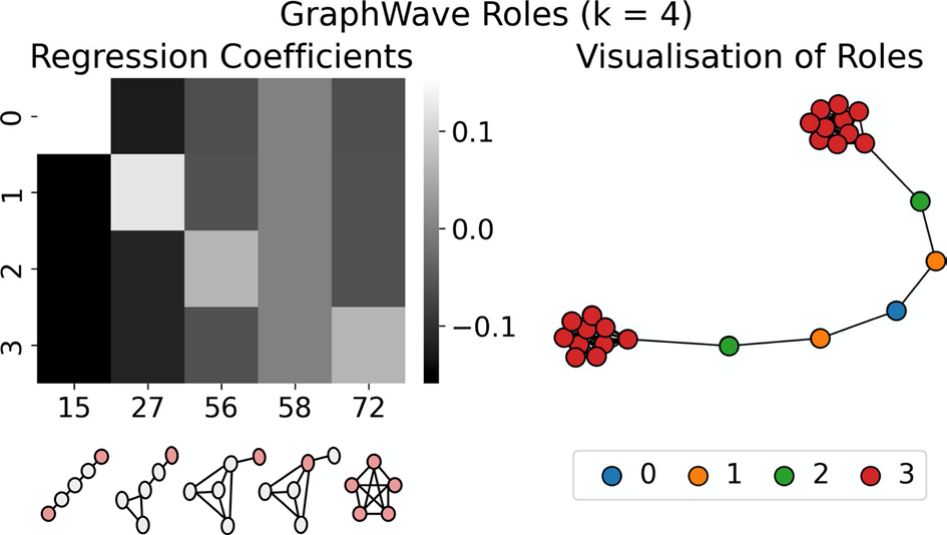
Structural explanation for role discovery via *k*-means clustering of *GraphWave* embeddings $(k = 4)$

The *GraphWave* and *Struc2Vec* methods identify different sets of roles at $k = 4$. Figures 6 and 7 explain the structures specific to these roles and can be used to understand the differences between the two groupings. For example, it is apparent from the visualisations that *Struc2Vec* role 1 and *GraphWave* role 2 refer to the same structures—nodes adjacent to large cliques—that were identified as role 2 in the $k = 5$ clustering. Further, from the $k = 5$ clustering, roles 0 and 1 are unchanged in the *GraphWave* $k = 4$ model, whereas they are combined to give a single role in the *Struc2Vec* $k = 4$ case. Conversely, *Struc2Vec* roles 2 and 3, represent roles 3 and 4 from the barbell graph, while the *GraphWave* approach is unable to distinguish between these structures; *GraphWave* role 3 represents all clique nodes and does not discriminate the ‘gateway’ node in the clique. This is evident in the explanation in Fig. 7 as the parameters for Orbit 58 are all 0.

**Fig. 8:**
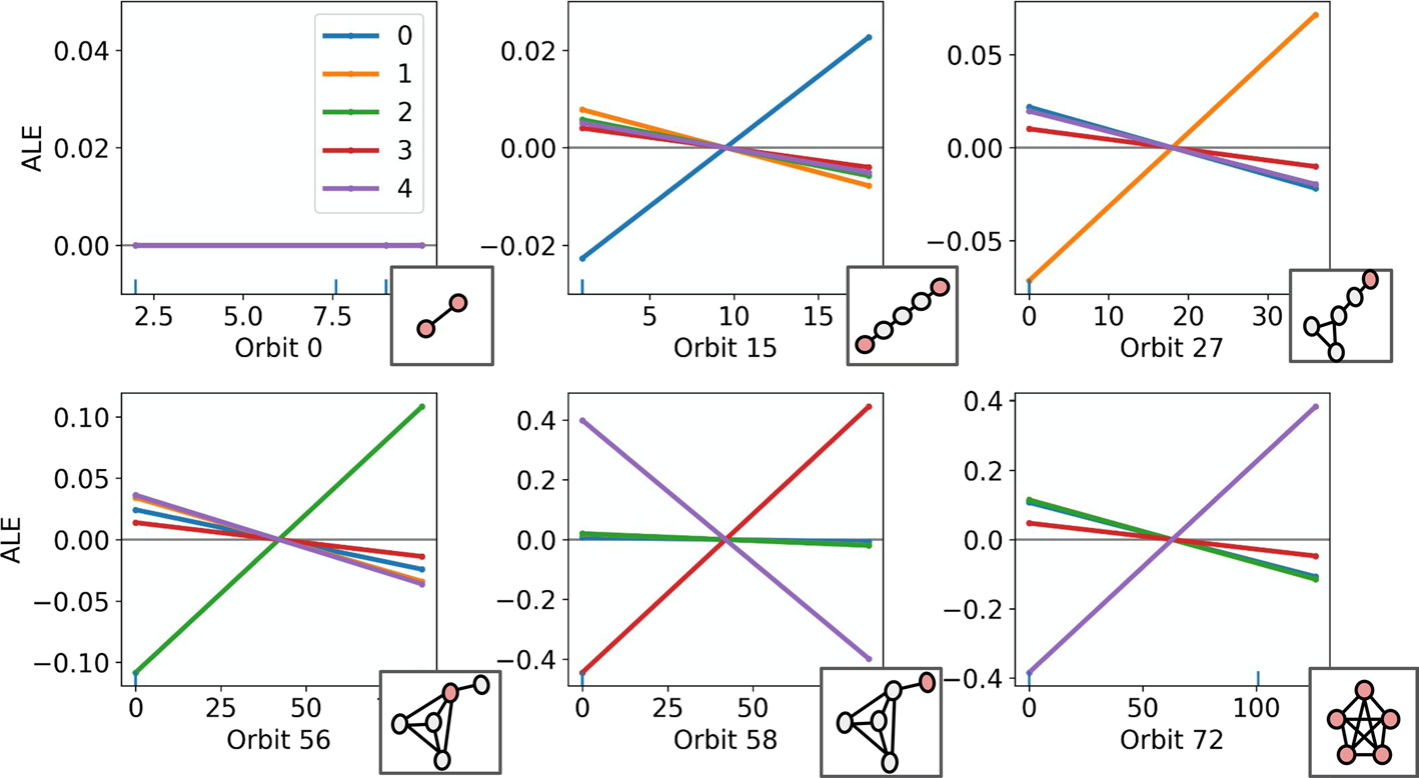
Accumulated Local Effects (ALE) plots illustrating the effect of different graphlet orbits on role assignments via a random forest surrogate model, for the 5 roles in the barbell graph. Each subplot represents a different feature/graphlet orbit and include the 5 discriminatory structures from the previous examples, and orbit 0 (node degree) for reference

In addition to explaining role assignments through simple, linear effects, it is useful (in the case of larger and more complex graphs), to understand role assignments with composite, non-linear effects. Such effects may be encountered when employing more expressive surrogate models, e.g., ensemble classifiers. To this end, we employ the Accumulated Local Effects (ALE) (Apley and Zhu 2020) plots in Fig. 8. The ALE plots explain the effects of different features on classification to the 5 barbell roles, according to a random forest classifier. Each ALE plot pertains to a feature and plots an effect for every class/role. Crucially, the ALE method considers the ‘local’ effect of features on the classification, meaning that effects are measured only *within* the distribution of the data. This behaviour is particularly important in the case of graph structures, since feature effects must be measured in a manner that obeys any correlations that exist between the orbit counts. As the effects in Fig. 8 appear largely linear, the explanations provided are very similar to those presented in Fig. 5. However, ALE plots are uniquely useful in interpreting non-linear effects, as will be seen in Sect. “Application”.

## Application

In this section we apply the SERD method described in Sect. “Methods” to extract and interpret sets of structural roles in a large, real-world citation network. In the case of each paper, we compute Rao-Stirling diversity scores (Stirling 2007) to indicate each article’s interdisciplinarity and thus explore the distributions of IDR scores assigned to papers in different roles. We identify a set of roles which has grouped papers according to their interdisciplinarity. Finally, we use graphlet orbit-based explanation to interpret the structure of these more interdisciplinary roles, thus allowing us to highlight certain citation structures that are specific to interdisciplinary research.

### Data

In order to discover the citation structures of interdisciplinary research, we require a large, dense citation network that contains research from a diverse set of disciplines. In addition, we require that each paper can be assigned to a subject category or discipline, according to an established taxonomy. We construct a novel citation network using Microsoft Academic Graph (Sinha et al. 2015) citation data from a seed set of journal papers. This set consist of samples of articles from Scopus indexed journals, stratified according to their All Science Journal Categories (ASJC). The graph contains samples of 1500 articles published between 2017 and 2018 in Scopus indexed journals with the ASJCs *‘Computer Science’, ‘Mathematics’, ‘Medicine’, ‘Chemistry’, ‘Social Sciences’, ‘Neuroscience’, ‘Engineering’*, and *‘Biochemistry, Genetics and Molecular Biology’*. We maximise the completeness of the graph by including all available referenced articles that are published in Scopus-indexed journals. In this manner, we produce a dense, multidisciplinary citation network, such that each article can be categorised according to the ASJC of the journal in which it was published. Later, these discipline categories can be used to compute article interdisciplinarity according to Rao-Stirling diversity of disciplines identified in both an articles citing and cited papers. In total, the citation graph contains 41,895 papers (nodes) and 129,159 citations (undirected edges).

### Role discovery

Following the experimental setup from Sect. “Demonstration/validation”, here for each article in the citation graph, we learn role embeddings using the same 4 approaches: (i) *Role2Vec* (Ahmed et al. 2019), (ii) *Struc2Vec* (Ribeiro et al. 2017), (iii) *RolX* (Henderson et al. 2012), (iv) *GraphWave* (Donnat et al. 2018), and we cluster each embedding space using *k*-means clustering for values of $k \in [2,19]$. As before, articles clustered according to their role embeddings represent a set of structural roles in the citation graph.

### Role interpretation

**Fig. 9:**
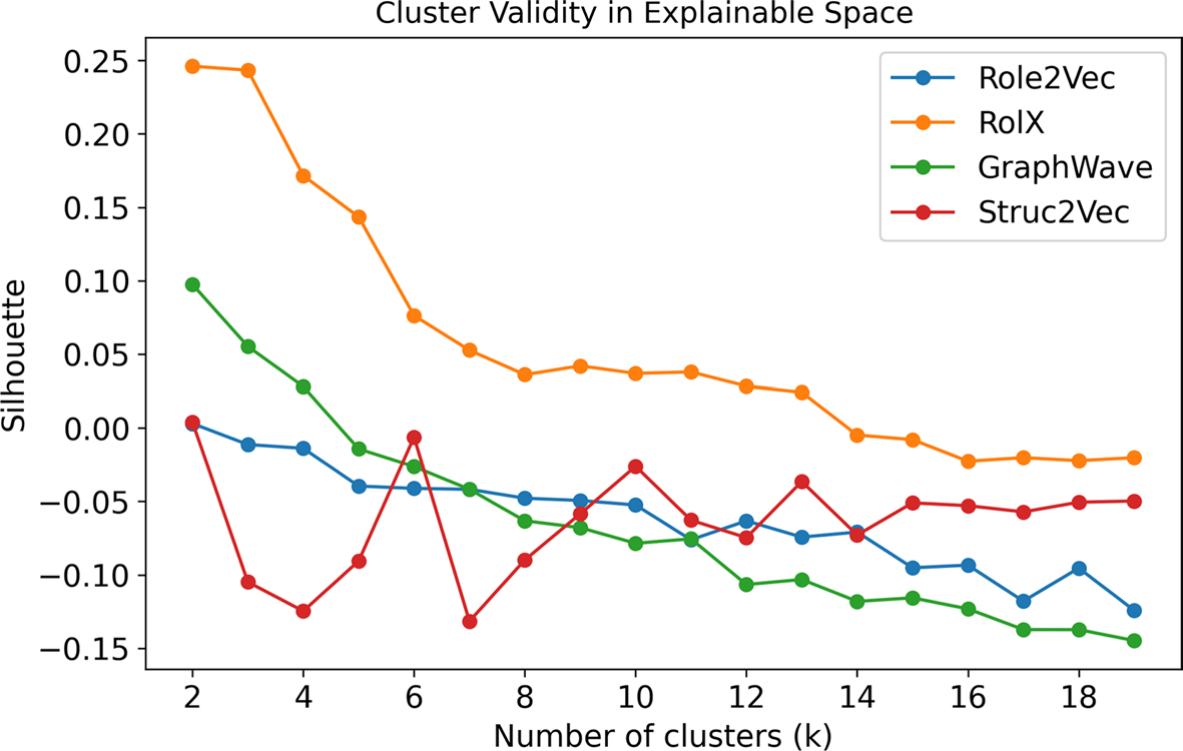
Silhouette score for *k*-means clusterings of the different embedding spaces. Scores are calculated according the clusterings when we describe each node by its bag-of-orbits vector, rather than its embedding

Figure 9 shows the cluster validity of the roles discovered according to the 4 role embedding methods. Each set of roles represents a *k*-means clustering of the embedding space, which is then transformed to the graphlet-orbit space, where we assess the validity of the clustering using the Silhouette measure as previously performed for the synthetic data in Sect. “Demonstration/validation” According to the Silhouette scores, we select 3 candidate roles to demonstrate interpretation and explanation: (i) *Struc2Vec* ($k = 6$) which is an outlier in the *Struc2Vec* roles and achieves an overlapping clustering, (ii) *RolX* ($k = 3$) which has the highest silhouette score for all approaches with $> 2$ clusters, and (iii) *GraphWave* ($k = 3$) which achieves a positive score.

We fit a random forest classifier to model role assignments according to the bag-of-orbits vectors for each node. That is, we learn a function $f:X_{g}\mapsto y_{role}$ for each of the candidate roles $y_{role}$. We choose a random forest classifier as we anticipate that structural roles may be non-linear in the graphlet-orbit space and may rely on combinations and interactions of features to model higher-order structures. As the orbit counts follow a power-law distribution, we log-transform all features in the graphlet-orbit space, after removing any zero values (due to the density of the graph, there are very few such entries).

Table 1 reports the 5 most informative features for each model according to permutation importance. In the case of the *Struc2Vec* ($k = 6$) roles, the overlap between clusters in the graphlet-orbit space is evident. The only informative features (with non-zero permutation importance) are small, local orbits—the approach is blind to deeper, more complex structures. The RolX ($k = 3$) approach, which best separates the nodes in the graphlet-orbit space, has grouped nodes according to larger, simple structures. The most informative orbits (15, 4, and 1) each refer to chains of varying length. Finally, *GraphWave* ($k = 3$) appears to have grouped the nodes according to more complex, higher-order structures. Many of the features important to role classification in the *GraphWave* case (27, 24, and 18) contain combinations of chains, stars, and cliques. Depending on the domain or application in which we employ role discovery, any one of these sets of roles may be the most valid or useful. However, without modelling the role assignments in the graphlet-orbit space, we are unable to understand which structures are being grouped in the discovered roles. We will now consider a single set of roles to demonstrate further explanation.

**Table 1:** Top 5 most important features for each surrogate model

	Struc2Vec ($k = 6$)	RolX ($k = 3$)	GraphWave ($k = 3$)
1	0 (0.111 ± 0.0005)	15 (0.113 ± 0.0016)	27 (0.022 ± 0.0006)
2	2 (0.010 ± 0.0001)	4 (0.007 ± 0.0002)	15 (0.014 ± 0.0002)
3	3 (0.006 ± 0.0001)	1 (0.006 ± 0.0003)	17 (0.013 ± 0.0006)
4	5 (0.002 ± 0.0001)	27 (0.005 ± 0.0002)	24 (0.013 ± 0.0004)
5	16 (0.000 ± 0.0000)	19 (0.004 ± 0.0003)	18 (0.013 ± 0.0004)

The orbits are ranked by permutation importance, with scores included in parentheses

### Role interpretation: GraphWave

**Fig. 10:**
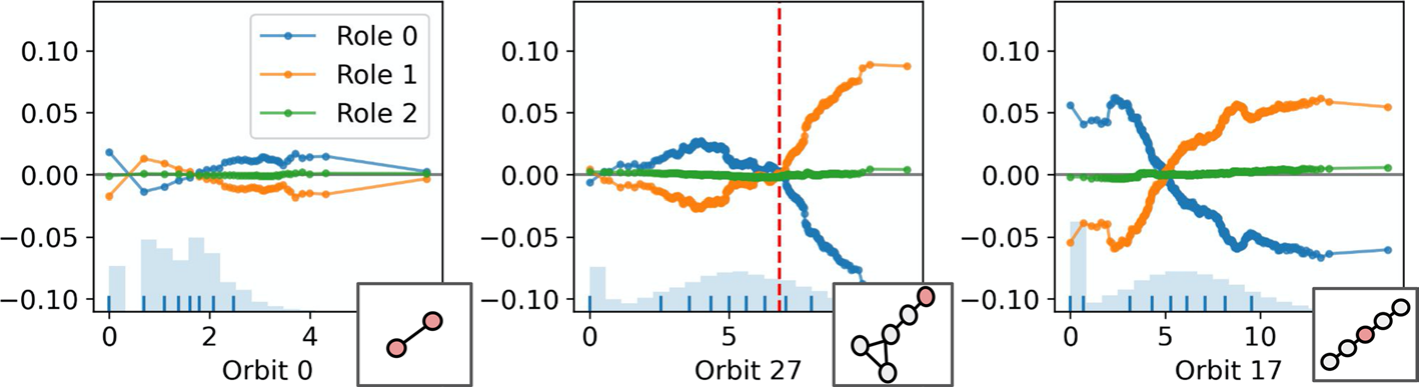
Accumulated Local Effect (Apley and Zhu 2020) plots for a surrogate model which classifies nodes to *GraphWave* roles according to graphlet orbit counts. The figure shows the effect of 3 features on role classification: orbits 27 and 17; the most important features as measured by permutation importance, and orbit 0 (node degree); which we include for reference. In the case of orbit 27, we highlight the maximum value of orbit 3 that was observed in the graph

**Fig. 11:**
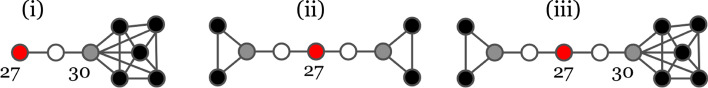
Higher order structures containing orbit 27. Here scenarios (i) and (ii) represent likely structures for nodes with high counts for orbit 27. When the count for orbit 27 exceeds a threshold, we infer structure (iii)

In the following sections, we explore the *GraphWave* $k = 3$ roles in greater detail. We ask ‘what are the structures specific to roles in a citation network, and what can we understand about the nature of the papers in these different roles?’ Specifically, we investigate the extent to which interdisciplinary research papers are structurally similar in their citation patterns, and highlight some of the important citation structures emblematic of interdisciplinary research. The *GraphWave* method clusters 35,136 papers into role 0, 16,453 papers into role 1, and 306 papers into role 2. Figure 10 shows Accumulated Local Effects (ALE) (Apley and Zhu 2020) plots for 3 features/orbits and their effect on classification to each of the 3 *GraphWave* roles. We illustrate the ALE of orbits 27 (the end of a chain adjacent to a clique) and 17 (the middle of a long chain) as two of the most important structures (according to permutation importance). We also include the ALE of orbit 0 (node degree) as a valuable reference, as it is useful to confirm that the roles are indeed separating nodes according to more complex features and not simply by the number of edges. The ALE plot for orbit 27 shows that for low-to-mid values of that orbit count, a node will be classified as role 0. However, if a node’s count for orbit 27 exceeds a threshold, it will be classified as role 1. We suppose two scenarios when a focal node’s count for orbit 27 (a chain adjacent to a clique) will become large: (i) the node is adjacent to a large community—each triangle in which the node at position 30 participates will increase the count; (ii) the node exists at the center of a barbell graphlet, i.e., on a longer chain between two or more communities communities. We illustrate these scenarios in Fig. 11. There should exist some threshold value for orbit 27, beyond which a node must exist on the chain between two communities. For example, if a focal node has a count for orbit 27 that is greater than the count of triangles (orbit 3) for the node at position 30, then the focal node must be adjacent to a second community (scenario (iii) in Fig. 11). This threshold will be represented by the greatest value of orbit 3 in the graph. We include this value for reference in Fig. 10. Beyond this threshold, a node is more likely to be classified in role 1. Accordingly, we conclude that a node that is on the end of a chain adjacent to a community will be assigned to role 0, while a node that exists on a bridge between two communities will be assigned to role 1.

**Fig. 12:**
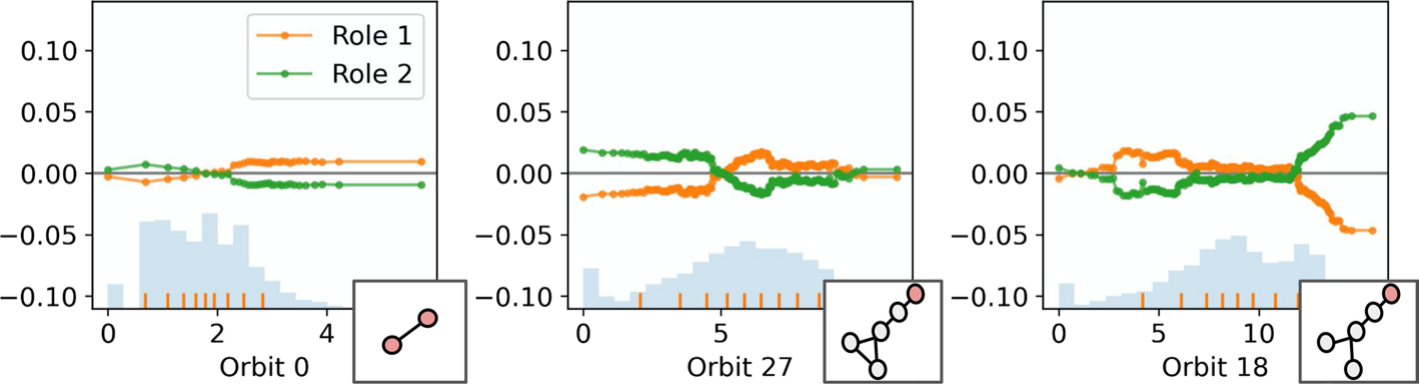
Accumulated Local Effect (Apley and Zhu 2020) plots for a surrogate model which classifies nodes to *GraphWave* roles 1 or 2 according to graphlet orbit counts. The figure plots the effect of 3 features: orbits 0; node degree and 27; previously the most important feature for the global model, and orbit 18; the most important feature in the surrogate model

In order to identify the structures specific to the smallest role (role 3), we can fit another surrogate model to only the nodes in cluster 1 and 2. ALE plots for this model are included in Fig. 12. In this case we find that orbit 27 does not meaningfully distinguish between the two roles. Instead, orbit 18 (the end of a chain adjacent to a star) is the most informative feature, and, for very high values of this orbit count, nodes will be assigned to role 2. Such nodes likely represent the centre of a bridge between large communities that are less densely connected (i.e., containing many open triads). We conclude this to be an important structure for role 3. As such, we have demonstrated how the SERD framework is capable of leveraging techniques from the field of XAI to reveal complex, higher-order motifs that represent the roles discovered in a large, real world network.

**Fig. 13:**
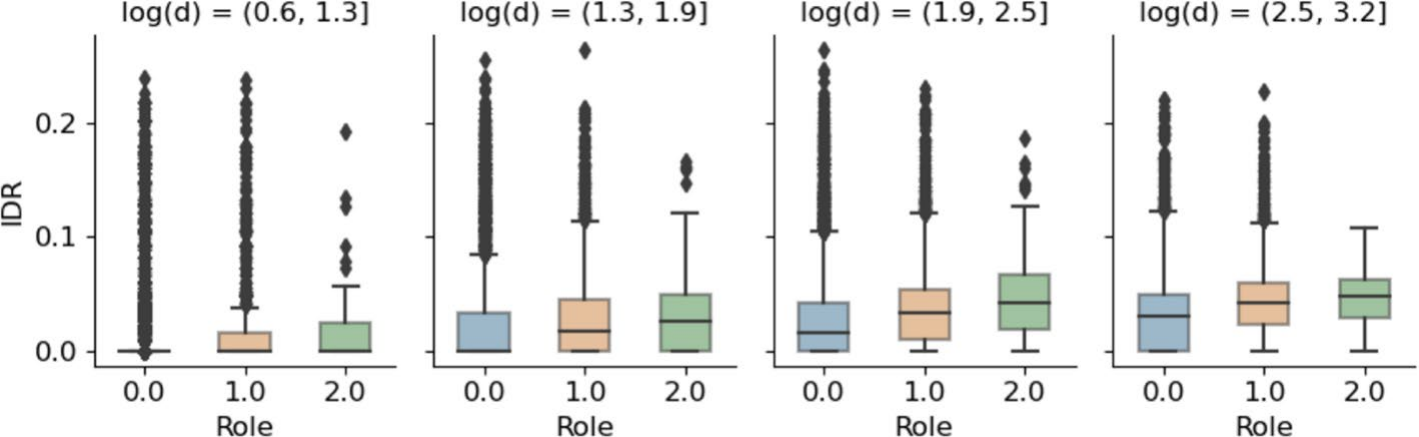
Interdisciplinarity scores (IDR) computed for papers in each of the 3 *GraphWave* clusters. IDR is computed as the Rao-Stirling (Stirling 2007) diversity of the research disciplines identified in an articles citing papers. We bin papers/nodes according to the log of their degree, and compare distributions within each bin

### Interdisciplinary roles

Finally, we examine whether the roles highlighted above are potential indicators of interdisciplinary research. Firstly, in Fig. 13 we examine the IDR scores for the papers assigned to each of the *GraphWave* roles. IDR is calculated according to the Rao-Stirling diversity of the ASJC categories identified in an article’s citing papers. As Rao-Stirling IDR scores may be biased according to the number of articles in the summation, we bin nodes by degree, and plot IDR distributions for each role, within each bin. Specifically, we log-transform the node degrees and group nodes into 10 bins of equal width, within which we plot IDR distributions for each role if the bin contains more than 50 papers from each role. According to these plots, we note that the structural roles identified by *GraphWave* have grouped papers into clusters with different IDR distributions. Even when we account for node degree (a potential bias of the Rao-Stirling IDR score), papers assigned to structural roles 1 and 2 have consistently greater IDR distributions than those assigned to role 0. We recall some of the structures that were identified by our approach as being important for roles 1 and 2: (1) a bridge between densely-connected communities, and (2) a bridge between large, sparsely-connected communities and conclude these to be important citation patterns associated with interdisciplinary research.

## Discussion and conclusions

Many modern methods for role discovery in graphs rely on node embeddings (Rossi et al. 2020). While these methods have previously been shown to be capable of grouping nodes into known roles (e.g., in synthetic graphs or transport networks (Ribeiro et al. 2017; Donnat et al. 2018)), it remains unclear how roles could be understood or validated when applied to graphs with unknown roles. In this work we proposed SERD (Surrogate Explanation for Role Discovery), a framework for interpreting a set of discovered roles using graphlets and orbits. We leveraged methods from the field of explainable AI to explore the subgraph structures that are specific to discovered roles. We first used a small synthetic graph to illustrate the workflow of the proposed framework, before demonstrating its application to a real-world citation network, where we identified important structures specific to interdisciplinary research. It is evident in our analysis that different role discovery methods learn different sets of structural roles. In certain applications many or all of these clusterings may be valid, but it is critical that we can compare the roles discovered by different methods. While our framework is general, and applicable to explanation and validation in all role discovery tasks, we highlighted the particular utility of structural role embeddings in mapping interdisciplinary research, and the benefits of employing our proposed framework in that context to understand the outputs of such embedding methods.

For the task of further identifying and mapping IDR interactions, structural paper embeddings could be augmented by considering additional, non-structural information, such as article or abstract text. This could provide a richer paper representation, without imposing a predefined or static disciplinary classification on the graph. There is also scope for improving upon our proposed framework. Many *model-agnostic* approaches have been developed for explaining surrogate models (Molnar 2020), which could be applied to interpret the role assignments in the graphlet-orbit space. For example, second-order effects of pairs of features can be calculated in a similar manner to the ALE analysis that we considered in this paper (Apley and Zhu 2020). Combinations of graphlets could be highly effective in modelling higher-order, more complex graph structures.

## Data Availability

An archive of the relevant metadata for both case studies will be made available online before any final publication.

## References

[CR1] Abramo G, D’Angelo CA, Zhang L (2018). A comparison of two approaches for measuring interdisciplinary research output: the disciplinary diversity of authors vs the disciplinary diversity of the reference list. J Inform.

[CR2] Adadi A, Berrada M (2018). Peeking inside the black-box: a survey on explainable artificial intelligence (XAI). IEEE Access.

[CR3] Ahmed NK, Rossi RA, Lee JB, Willke TL, Zhou R, Kong X, Eldardiry H (2019) role2vec: role-based network embeddings. In: Proceedings of DLG KDD, pp 1–7

[CR4] Apley DW, Zhu J (2020). Visualizing the effects of predictor variables in black box supervised learning models. J R Stat Soc Ser B (Stat Methodol).

[CR5] Breiman L (2001). Random forests. Mach Learn.

[CR7] Cunningham E, Greene D (2022) Assessing network representations for identifying interdisciplinarity. arXiv preprint arXiv:2203.12455

[CR8] Cunningham E, Greene D (2023) The structure of interdisciplinary science: uncovering and explaining roles in citation graphs. In: Complex Networks and Their Applications XI: Proceedings of the eleventh international conference on complex networks and their applications, vol 1, pp 364–376. Springer, Berlin

[CR6] Cunningham P, Harrigan M, Wu G, O’Callaghan D (2013). Characterizing ego-networks using motifs. Netw Sci.

[CR9] Dehghan A, Siuta K, Skorupka A, Betlen A, Miller D, Kamiński B, Prałat P (2023) Unsupervised framework for evaluating structural node embeddings of graphs. Preprint 1, 16

[CR10] Donnat C, Zitnik M, Hallac D, Leskovec J (2018) Learning structural node embeddings via diffusion wavelets. In: Proceedings of 24th ACM SIGKDD international conference on knowledge discovery & data mining, pp 1320–1329

[CR11] Friedman JH (2001) Greedy function approximation: a gradient boosting machine. Ann Stat 1189–1232

[CR12] Henderson K, Gallagher B, Eliassi-Rad T, Tong H, Basu S, Akoglu L, Koutra D, Faloutsos C, Li L (2012) Rolx: structural role extraction & mining in large graphs. In: Proceedings of 18th ACM SIGKDD international conference on knowledge discovery and data mining, pp 1231–1239

[CR13] Hočevar T, Demšar J (2017). Combinatorial algorithm for counting small induced graphs and orbits. PLoS One.

[CR14] Jain AK, Dubes RC (1988). Algorithms for clustering data.

[CR15] Jin J, Heimann M, Jin D, Koutra D (2021). Toward understanding and evaluating structural node embeddings. ACM Trans Knowl Discov Data (TKDD).

[CR16] Lombrozo T (2007). Simplicity and probability in causal explanation. Cognit Psychol.

[CR17] Miller T (2018) Contrastive explanation: a structural-model approach. Knowl Eng Rev 3

[CR18] Milo R, Shen-Orr S, Itzkovitz S, Kashtan N, Chklovskii D, Alon U (2002). Network motifs: simple building blocks of complex networks. Science.

[CR19] Milojević S (2020). Practical method to reclassify Web of Science articles into unique subject categories and broad disciplines. Quant Sci Stud.

[CR20] Molnar C (2020) Interpretable machine learning. Lulu.com

[CR21] Moreno JL (1934) Who shall survive?: A new approach to the problem of human interrelations

[CR22] Porter A, Chubin D (1985). An indicator of cross-disciplinary research. Scientometrics.

[CR23] Porter A, Rafols I (2009). Is science becoming more interdisciplinary? measuring and mapping six research fields over time. Scientometrics.

[CR24] Pržulj N (2007). Biological network comparison using graphlet degree distribution. Bioinformatics.

[CR25] Rafols I, Meyer M (2010). Diversity and network coherence as indicators of interdisciplinarity: case studies in bionanoscience. Scientometrics.

[CR26] Ribeiro LF, Saverese PH, Figueiredo DR (2017) struc2vec: learning node representations from structural identity. In: Proceedings of 23rd ACM SIGKDD international conference on knowledge discovery and data mining, pp 385–394

[CR27] Rossi RA, Ahmed NK (2014). Role discovery in networks. IEEE Trans Knowl Data Eng.

[CR28] Rossi RA, Jin D, Kim S, Ahmed NK, Koutra D, Lee JB (2020). On proximity and structural role-based embeddings in networks: Misconceptions, techniques, and applications. ACM Trans Knowl Discov Data (TKDD).

[CR29] Rosvall M, Bergstrom CT (2008). Maps of random walks on complex networks reveal community structure. PNAS.

[CR30] Rousseeuw PJ (1987). Silhouettes: a graphical aid to the interpretation and validation of cluster analysis. J Comput Appl Math.

[CR31] Sadler S, Greene D, Archambault D (2021) Selecting informative features for post-hoc community explanation. In: International conference on complex networks and their applications, pp 297–308. Springer, Berlin

[CR32] Shen Z, Chen F, Yang L, Wu J (2019). Node2vec representation for clustering journals and as a possible measure of diversity. J Data Inf Sci.

[CR33] Sinha A, Shen Z, Song Y, Ma H, Eide D, Hsu B-J, Wang K (2015) An overview of microsoft academic service (MAS) and applications. In: Proceedings of 24th international conference on world wide web, pp 243–246

[CR34] Stirling A (2007). A general framework for analysing diversity in science, technology and society. J R Soc Interface.

[CR35] Van Noorden R (2015). Interdisciplinary research by the numbers. Nature.

[CR36] Wagner CS, Roessner JD, Bobb K, Klein JT, Boyack KW, Keyton J, Rafols I, Börner K (2011). Approaches to understanding and measuring interdisciplinary scientific research (IDR): a review of the literature. J Informetr.

[CR37] Yaveroğlu ÖN, Malod-Dognin N, Davis D, Levnajic Z, Janjic V, Karapandza R, Stojmirovic A, Pržulj N (2014). Revealing the hidden language of complex networks. Sci Rep.

